# *PHF6* Mutations in Hematologic Malignancies

**DOI:** 10.3389/fonc.2021.704471

**Published:** 2021-07-26

**Authors:** Jason H. Kurzer, Olga K. Weinberg

**Affiliations:** ^1^ Department of Pathology, Stanford University School of Medicine, Stanford, CA, United States; ^2^ Department of Pathology, UT Southwestern, Dallas, TX, United States

**Keywords:** PHF6, leukemia, tumor suppressor, T-ALL, AML

## Abstract

Next generation sequencing has uncovered several genes with associated mutations in hematologic malignancies that can serve as potential biomarkers of disease. Keeping abreast of these genes is therefore of paramount importance in the field of hematology. This review focuses on *PHF6*, a highly conserved epigenetic transcriptional regulator that is important for neurodevelopment and hematopoiesis. *PHF*6 serves as a tumor suppressor protein, with *PHF6* mutations and deletions often implicated in the development of T-lymphoblastic leukemia and less frequently in acute myeloid leukemia and other myeloid neoplasms. *PHF6* inactivation appears to be an early event in T-lymphoblastic leukemogenesis, requiring cooperating events, including *NOTCH1* mutations or overexpression of TLX1 and TLX3 for full disease development. In contrast, *PHF6* mutations tend to occur later in myeloid malignancies, are frequently accompanied by *RUNX1* mutations, and are often associated with disease progression. Moreover, *PHF*6 appears to play a role in lineage plasticity within hematopoietic malignancies, with *PHF6* mutations commonly present in mixed phenotype acute leukemias with a predilection for T-lineage marker expression. Due to conflicting data, the prognostic significance of *PHF6* mutations remains unclear, with a subset of studies showing no significant difference in outcomes compared to malignancies with wild-type *PHF6*, and other studies showing inferior outcomes in certain patients with mutated *PHF6.* Future studies are necessary to elucidate the role *PHF6* plays in development of T-lymphoblastic leukemia, progression of myeloid malignancies, and its overall prognostic significance in hematopoietic neoplasms.

## Introduction

**Figure 1:**
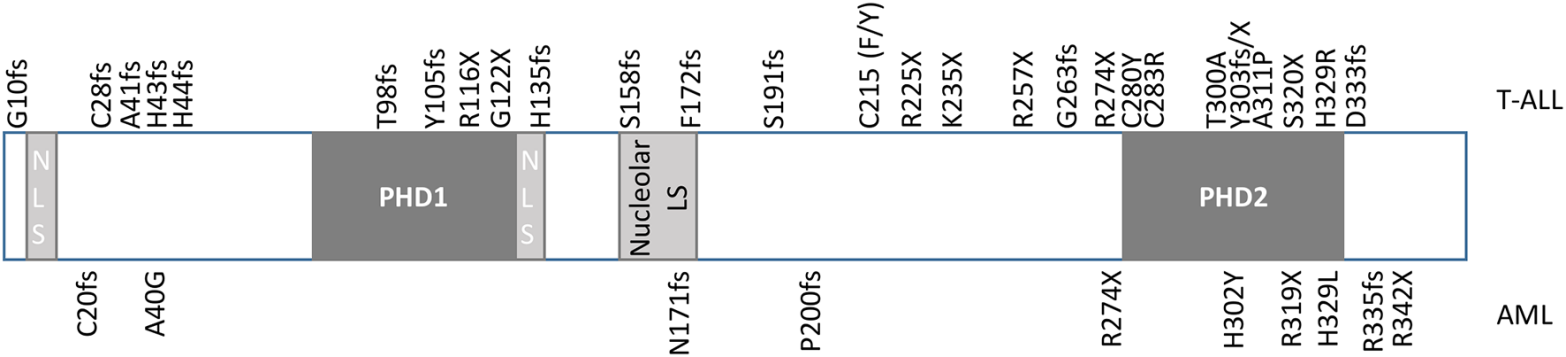
Diagram of *PHF6*. *PHF6* has two imperfect PHD-like zinc finger domains (PHD1 and PHD2), two nuclear localization signals (NLS), and one nucleolar localization signal. Mutations originally identified in T-ALL by Van Vlierberghe et al. (4) are identified at the top of the diagram, whereas mutations originally identified in AML by Van Vliergerghe et al. (21) are identified at the bottom of the diagram.

Plant homeodomain (PHD) finger proteins consist of a family of epigenetic regulators that bind to a variety of targets, including both post-translationally modified and unmodified histones (1). The PHD finger protein, homeodomain finger protein 6 (*PHF6*), is a highly conserved, 365 amino acid, 41kDa protein, that was first identified in the X-linked neurodevelopmental disorder, Börjeson-Forssman-Lehmann syndrome (BFLS) (2, 3). *PHF6* contains two imperfect PHD-like zinc finger domains, two nuclear localization signals as well as a nucleolar localization sequence (**Figure 1**) (2, 4–6). Expression of PHF6 is found in almost all tissues, with particularly high expression in the brain/developing central nervous system as well as in all hematopoietic subpopulations (high levels in CD34+ precursor cells and B-cells; low levels in NK-cells and monocytes) implicating a role for *PHF*6 in a variety of functions including neurogenesis and hematopoiesis (2, 7, 8).

## Function of *PHF6*

Supporting a fundamental role for *PHF6* in general development, *PHF6-*homozygous knockout mice die perinatally (9). However, scientists have also developed various knockout models in mice to explore the role of *PHF*6 in hematopoiesis (8, 10–13). McRae et al. showed that germline deletion of murine *PHF6* is lethal in males whereas heterozygous females survived to adulthood (10). Using *Phf6^lox/Y^;Tie-cre^Tg/+^* male mice with a *Phf6*-null mutation in hematopoietic and endothelial cells, McRae et al. showed an increase in Lin^-^c-Kit^+^Sca-1^+^ (LSK) stem and progenitor cell-enriched populations, specifically the heterogeneous progenitor cell population (HPC-1), with an enrichment in cycling forms (10). Inactivation of *PHF6* at various stages of development results in increased embryologic proliferative properties of hematopoietic stem cells, but also an increased ability for *PHF6-*deficient neonatal and adult HSCs to repopulate the bone marrow in serial transplant assays (10–13). Studies suggest that this enhanced proliferative ability of *PHF6-*deleted HSCs results, at least in part, from IFN-α signaling, the inhibition of TNFα-associated growth suppressing genes, and perhaps upregulation of JAK1 signaling (10–12).

Wendorff et al. similarly showed that conditional knockout of *PHF6* displayed expansions of total immature hematopoietic LSK cells, but also showed increased multipotent MPP2, and MPP3 populations in 8-week-old mice, and no differences in the numbers of more mature myeloid progenitors, uncommitted lymphoid progenitors, or B-cell precursors (11). However, conditional knockout of *PHF6* does show a mild reduction in double-negative DN2 and DN3 thymic progenitors and decreased numbers of peripheral blood CD4 and CD8 positive T-cells in 8-week-old mice (10, 11, 13). Moreover, knockdown studies in cord blood and thymus-derived hematopoietic precursors showed that *PHF6* loss fosters a preferential differentiation of B-lymphocytes, reduced erythroid development, and accelerated T-cell development *via* downregulation of *NOTCH1* (8).

Like other PHD finger proteins, *PHF6* functions in chromatin-mediated regulation of gene expression. It is capable of binding double-stranded DNA, but not histones, *in vitro via* its atypical PHD2 domain (14). Co-immunoprecipitation experiments using antibodies to *PHF6*, reveal it interacts with constituents of the nucleosome remodeling deacetylase (NuRD) complex, including CHD_4_, HDAC_1_, and Rbb_4_ (6, 15). The NuRD complex is a major ATP-dependent chromatin remodeling complex, implicated in nucleosome positioning and both repressing and activating genes involved in embryonic development (16). PHF6 has additionally been shown to bind to the PAF1 transcription elongation complex, regulating Neuroglycan C/Chondroitn sulfate proteoglycan 5 (NGC/CSPG5) and ultimately neuronal migration in murine cerebral cortex development (17). One consequence of *PHF6*-mediated transcription regulation includes the modulation of levels of the RNA Pol I preinitiation complex activator, upstream binding factor (UBF), thereby suppressing UBF-mediated rRNA transcription (5, 18).

Knockdown studies of PHF6 suggest a tumor suppressor role for the protein, as PHF6 deficiency in HeLa cell lines resulted in increased UBF protein levels, and increased DNA damage at the rDNA locus (5). Deficiency of PHF6 results in the formation of DNA-RNA hybrid (R-loops) and increased R-loop-dependent rDNA damage (5). Moreover, knockdown of *PHF6* interferes with the G2 checkpoint recovery of U2OS cells, leading to decreased DNA repair in response to ionizing radiation (19). These data implicate a tumor suppressor role for PHF6 resulting from regulating DNA damage response.

## *PHF6* and Hematologic Disease

Despite the discovery of *PHF6 via* its role in BFL syndrome and its prominent role in neurogenesis, mutations of *PHF6* have only been identified to date in hematologic malignancies (20). The first and most well-documented hematologic malignancy harboring mutations of *PHF6* is T-lymphoblastic leukemia (T-ALL), with fewer cases identified in acute myeloid leukemia (AML), and rarer cases identified in pre-malignant clonal hematopoiesis (4, 21–23). Mutations in hematologic malignancies include deletion, missense, frameshift, and nonsense mutations, which span the whole coding region, with missense mutations concentrated in the PHD2 domain (**Figure 1**) (4, 21, 24). The ultimate effect of these mutations is to inhibit PHF6 function or deplete its levels, and as such, provide supporting evidence that this protein serves as a tumor suppressor. Indeed, a patient with BFLS was noted to develop T-ALL (25).

### T-Lymphoblastic Leukemia

The *PHF6* locus is one of the most frequently mutated genes in T-lymphoblastic leukemia (T-ALL). Inactivating mutations of *PHF6* have been identified in 5-16% of pediatric and 19-40% of adult patients with T-ALL and ~25% of adults with T-lymphoblastic lymphoma (T-LBL) with some groups identifying an association with *NOTCH1* mutations (67%-84.6% of *PHF6* mutated/deleted T-ALL with *NOTCH1* mutations versus 39.8% *PHF6* WT) (4, 26–38). Copy number alterations of *PHF6* in pediatric T-ALL has been reported to be between 13-14% (39, 40). The location of *PHF6* on chrX26.2 lead researches to speculate that such mutations may at least partially explain the 2-3 fold increased incidence of T-ALL in males, as *PHF6* mutations were originally found predominantly in male T-ALL patients (31.5% *vs* 2.6%) (4). Subsequent studies, however, have failed to show a gender preference (26–28, 33). Recently, *PHF6* mutations have additionally been found in up to 25% (3/12) of early T-cell precursor subtype of T-ALL (ETP), a form of T-ALL that frequently expresses myeloid-associated markers (41, 42).

The role *PHF6* plays in leukemogenesis is actively under investigation. Analyzing whole exome sequencing data from diagnostic and relapse leukemias, Wendorff et al. showed that somatic mutations of *PHF6* occur early in leukeomogenesis (11). Nevertheless, animal models have revealed that while *PHF6* mutations/deletions may be initial events, they are insufficient for tumor initiation without additional driver mutations. For example, Miyagi et al. did not observe leukemia development subsequent to serial transplantation of *PHF6*-deleted HSCs in mice (12). Likewise, no T-ALL tumors developed in a *PHF6* ^+/-^ zebrafish model (43).

Studies show that *PHF6* dysfunction cooperates with several other driver mutations. For example, inactivation of *PHF6* in hematopoietic progenitors has been reported to facilitate NOTCH1-induced T-ALL, potentially through increasing leukemia-initiating cells and development of a “leukemia stem cell transcriptional program” in lymphoblasts (11, 13). A study of 102 pediatric T-ALL cases in Taiwan showed that *PHF6* mutations frequently cooperate with *HOX11L2* overexpression and/or *WT1* mutations (44). In addition, *PHF6* mutations have been shown to be associated (at times in conjunction with *DNM2*) with T-ALLs that overexpress homeobox transcription factors, *TLX1* and *TLX3* (4, 32, 45). Indeed, ectopic expression of TLX3 in *PHF6-*deleted mice facilitated early onset leukemia and a *hTLX1;PHF6^+/-^* zebrafish model demonstrated fully penetrant early-onset leukemia development, underscoring the role of cooperation between these mutations in leukemogenesis (10, 43). Other associations include those with mutations in X-linked genes, *USP9X* and *MED1* as well as with *IL7R-JAK* pathway genes, *WT1, PTPN2* deletions, and *HOX11L2* overexpression (33, 34, 44, 46). In the setting of ETP, *PHF6* mutations frequently occur with mutations in *EZH2*, *EED*, and *SUZ12* (41). Finally, a network of miRNAs, including miR-19b, miR-20a/93, miR-26a, miR-92, and miR-223, have been shown to target multiple PHF6 and other T-ALL-associated tumor suppressors, and thus promote leukemia (47).

*PHF6* has additionally been shown to interact with LMO2 to bind DNA *via* the LMO2/TAL1/LDB/GATA2 complex in T-ALL cell lines (48). The PHF6/LMO2/TAL1/LDB/GATA2 complex was shown to bind at DNA segments associated with hematopoietic or lymphoid organ development, hematopoiesis, as well as T-cell activation and differentiation (48).

While sample sizes are small, patients with T-ALL harboring *PHF6* mutations tend to be older, have been demonstrated to have lower white blood cell counts than other T-ALL patients, as well as lower hemoglobin and platelet levels, splenomegaly/lymphadenopathy, and have blasts with a tendency to express CD13 (26, 29, 37). Genomic analysis of matched diagnosis, germline (remission) and relapse DNA samples from 46 T-ALL cases reveals that *PHF6* alterations are found commonly at diagnosis, and persist at relapse (49). While mutations in *PHF6* have been implicated in increased resistance to prednisolone in T-ALL cell lines, the majority of studies have shown no correlation with *PHF6* mutations and overall survival in patients with T-ALL, and a potential favorable prognosis associated with T-LBL (4, 26, 27, 29, 50, 51). Nevertheless, one study of pediatric T-ALL found *PHF6* mutations/deletions predict an inferior overall survival upon multivariate analysis (44). Another study of Chinese adults found that the co-existence of *PHF6* and *NOTCH1* mutations in T-ALL conferred a shorter event-free survival and a poor prognosis (37). Further study is therefore required to assess the true prognostic significance of *PHF6* mutations in T-ALL.

### Myeloid Neoplasms

The largest study of *PHF6* mutations in myeloid malignancies involved targeted sequencing of 1760 cases with myeloid neoplasms (24). This study revealed 54 patients with 62 somatic mutations of *PHF6* (24). With regard to disease burden, the percentage of blasts in the bone marrow tended to be higher in patients with myeloid neoplasms harboring *PHF6* mutations (24). As for cytogenetics, abnormal karyotypes showed no significant predilection for *PHF6* mutations, although +8, t(8;21), and complex karyotypes were abnormalities most often identified (24). At the molecular level, co-mutated genes associated with *PHF6* mutations included *RUNX1*, *U2AF1*, *SMC1A*, *ZRSR2*, *EZH2*, and *ASXL1*, whereas *PHF6* was found to be mutually exclusive with *SF3B1* (24). In contrast to T-ALL, mutations of *PHF6* tend to occur later in disease evolution, sometimes with different mutations in parallel clones (24).

#### Acute Myeloid Leukemia

Inactivating somatic *PHF6* mutations have been found in ~2-3% of AMLs (20, 21, 24, 28, 52, 53). Similar to T-ALL, a male predominance was initially reported but not further substantiated for AML identified with *PHF6* mutations (21, 24, 53). With respect to AML subtypes, *PHF6* mutations are found in 15% of AML with inv(3)(q21q26.2)/t(3;3)(q21;q26.2) and 15.4% of cases of AML with myelodysplasia related changes (MRC) (24, 54). Interestingly, a case report of AML with MRC harboring a *P2RY8-CRLF2* fusion was found to have gained a *PHF6* mutation upon transformation to AML, suggesting a potential role for *PHF6* in the transition of MDS to AML (55). Further evidence for *PHF6* mutations acquired secondarily and leading to progression of myeloid neoplasms was found in patients with germline mutations of *RUNX1*, where *PHF6* mutations were implicated in the transition to MDS in one patient and the transition to AML in the other (56, 57). Interestingly, *PHF6* has been shown to frequently co-occur with *RUNX1* in AML (58).

Despite *PHF6* mutations leading to inactivation of the protein, an analysis of *PHF6* expression levels in AML regardless of mutation status revealed that PHF6 protein levels are higher in patients with AML than normal controls, a finding seemingly at odds with its role as a tumor suppressor (59). Moreover, increased PHF6 levels correlated with an increased percentage of blasts, with a possible correlation with CD34 positivity (59). Decreased PHF6 protein expression correlated with longer overall survival than those with high expression levels (2 years versus 6 months) (59).

A study of 318 pediatric patients with *de novo* AML identified *PHF6* mutations in 6 (2%) cases with FAB subtypes of M0, M1, and M2 (53). The median age for this group was 12.6 years (versus 9.5 in wild type *PHF6 AML*), with 4 of 6 succumbing to the disease. Co-genetic abnormalities included *RUNX1/RUNX1T1* translocations, *NUP98/KDM5A* translocations, and mutations in *WT1*, *RAS*, *ETV6*, *TET2*, *IDH1*, and *BCORL1* (53). Measuring the expression level of PHF6 showed decreased PHF6 levels in patients with mutations compared to M0, M1, and M2 AML subtypes with wild-type *PHF6*, again supporting a tumor suppressor role for *PHF6* and providing at least some genetic context to the results found by Mousa et al. above (53, 59).

A study of 398 patients with AML younger than 60 years of age revealed *PHF6* to be associated with decreased overall survival in patients with intermediate-risk AML with wild-type FLT3-ITD (60). This implicates *PHF6* mutations as a potential prognostic marker to be used in intermediate-risk AML, although it should be noted that this finding has not been replicated by others (24). Interestingly, subdividing AML with MRC cases that are associated with complex karyotypes into typical (those harboring 5q, 7q and/or 17p abnormalities) and atypical (those without these abnormalities) shows that AML with atypical complex karyotypes tend to have *PHF6* mutations more frequently, *TP53* mutations less frequently, be younger, have a higher WBC and blast percentage, and higher complete remission and overall survival rates (61).

#### Myelodysplastic Syndrome

Animal models suggest a possible role for *PHF6* mutations in myelodysplastic syndrome (MDS), as aged mice with knocked-out *PHF6* exhibit megakaryocytic dysplasia and associated decreased platelet counts as well as extramedullary hematopoiesis (13). Nevertheless, *PHF6* mutations are relatively rare (~3%) in MDS (24, 62–64). The limited data indicate that *PHF6* mutations are found most frequently (5.3% of MDS cases) in the high-grade subtypes (MDS with excess blasts) (24). *PHF6* mutations tended to show low variant allele frequencies and acquisition in sub-clonal populations (24). The most frequent co-mutations were seen in ASXL1, RUNX1, TET2, and DNMT3A (64). A study of 21 MDS patients harboring *PHF6* mutations revealed 61.9% had normal karyotypes and no patients had complex karyotypes (64).

#### Myeloproliferative Neoplasms

*PHF6* mutations are rarely identified in myeloproliferative neoplasms (MPN) (0.7%), occurring in only 1.6% of chronic myelogenous leukemia (24). A screen of 81patients with CML in myeloid blast crisis identified 2 male patients with *PHF6* mutations, with at least one patient showing no *PHF6* mutations in the preceding chronic phase (65). This finding raises the possibility that, similar to MDS, the accumulation of *PHF6* mutations might mediate progression of the disease. Relatedly, a review of 22 patients with *PHF6* mutations in myeloproliferative neoplasms at three institutions revealed an enrichment in cases with increased fibrosis and/or blast crisis (66). Other than JAK2, the most common co-mutations in these MPNs were *ASXL1*, and *TET2* with a median of 2.5 non JAK2 co-mutated genes (66).

With respect to mixed myelodysplastic syndrome/myeloproliferative neoplasm cases, *PHF6* mutations were seen in 4.7% of CMML patients (24). To date, no effect on survival has been seen in *PHF6-*mutation associated CMML patients (64).

### B-Lymphoblastic Leukemia

Despite their prevalence in T-ALL, *PHF6* mutations have only rarely been identified in B-lymphoblastic leukemia (4, 41, 67). 50% of the rare *MEF2D-*rearranged B-ALL was found to have *PHF6* mutations (67). Intriguingly, use of a retrovirally-expressed shRNA screening library into a B-lymphobastic leukemia cell line revealed that knockdown of PHF6 levels inhibited cell growth and leukemia growth in transplanted models (68). Indeed, CRISPR-Cas9-mediated deletion of *PHF6* in a murine *BCR-ABL1^+^; p19^-/-^; mCherry^+^* B-ALL cell line resulted in delayed tumor formation after injection in immunocompetent mice compared to wild-type B-ALL cells (69). Moreover, *PHF6* KO B-ALL cells induced a malignancy closer in presentation to lymphoma than leukemia, with tumor cells showing reduced expression of CD19 and B220 and B-cell development genes (e.g., *Cd74*, *IL4ra*, *Lyn*, *Ly86*, and *BLK*), and upregulation of CD4 and T-cell signal transduction genes (69). These data therefore implicate PHF6 mutations as lineage specific with respect to tumorigenesis, and even implicate mutation of *PHF6* as a potential mediator of lineage plasticity in hematopoietic neoplasms.

### Acute Leukemia of Ambiguous Lineage

The association of *PHF6* mutations with leukemias of ambiguous lineage further supports a role for these mutations in lineage plasticity. An analysis of 29 mixed phenotype acute leukemia cases at Memorial Sloan Kettering Cancer Center revealed *PHF6* (23%) and *DNMT3A* (23%) as the most common recurrent mutations (70). Mutations in *PHF6* and *DNMT3A* are mutually exclusive in MPAL, correlate with T-lineage marker expression (83% and 100%, respectively), and have higher relapse at 2 years (58% and 79%, respectively) compared to MPAL lacking these mutations (70).

In addition to T/M cases, Xiao et al. identified *PHF6* mutations in patients with T/B/M and T/B phenotypes (70). Similarly, Getta et al. found *PHF6* mutations in 3 of 16 MPAL patients with at least 2 of 3 patients of the MPAL, NOS subtype (B/T or B/T/M) (71). Furthermore, a review of 9 patients from multiple institutions identified *PHF6* mutations in 5 patients with B/T MPAL (56%) (72).

Similar to T-ALL, *PHF6* mutations are believed to be early events in MPAL, as Xiao et al. found every blast population isolated from selected cases showed nearly 100% VAF (70). Interestingly, *PHF6*-associated MPAL correlates with younger patients, higher hemoglobin and higher platelet values (70, 72).

Bond et al. performed an analysis of the transcriptional program of AMLs and T-ALLs and identified an expression program at the interface of these two diseases (73). Comparing T-ALL to T-ALL-like AMLs and AML-like T-ALLs, it was found that all T-ALLs with *PHF6* mutations were accompanied by *NOTCH1* mutations, whereas 3/5 *PHF6* mutated interface cases lacked *NOTCH1* mutations (73).

Interestingly, a study of acute undifferentiated leukemias found *PHF6* mutations in 7/16 cases of AUL, whereas only 1/25 cases of minimally differentiated AML harbored these mutations (74). Nevertheless, in the limited study, no clinical outcome differences were seen between the two groups (74).

## Discussion

**Figure 2:**
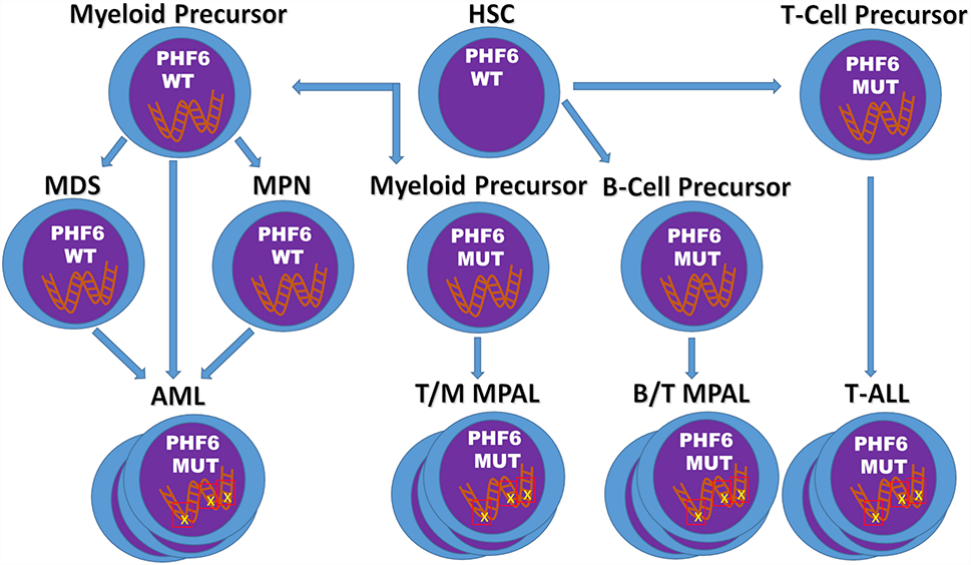
Model of *PHF6* in Hematopoietic Malignancies. The timing and context of the acquisition of *PHF6* deletions/mutations appear to determine the fate of the resulting malignancy. In T-ALL, *PHF6* deletions/mutations arise early, but are insufficient for transformation into leukemia. In contrast, in myeloid neoplasms, *PHF6* deletions/mutations tend to develop later possibly leading to disease progression. Finally, acquisition of inactivating mutations of *PHF6* in myeloid or B-cell precursors may promote T-cell gene expression and eventual development of mixed phenotype acute leukemias.

The last two decades have elucidated a role for *PHF6* in neurodevelopment and hematopoiesis, and revealed it as a potent tumor suppressor with an exclusive tendency for hematologic malignancies (**Figure 2**). *PHF6* mutations are most common in T-ALL, and appear early in the disease course yet appear insufficient for leukemia development. Given the conflicting data regarding the prognostic significance of *PHF6* mutations in T-ALL, additional studies are necessary to clarify the role of *PHF6* inactivation in the disease course. It is likely that appropriately powered studies will need to assess its significance in specific patient cohorts.

In contrast to T-ALL, *PHF6* mutations are less frequent in myeloid malignancies. Of interest, these mutations are more frequently found later in the disease course at points of disease progression. Further work is therefore required to determine the mechanism by which *PHF6* pushes these neoplasms to a more aggressive disease, as well as to determine the overall prognostic significance of *PHF6* mutations in myeloid malignancies in general. If additional studies continue to show a role for *PHF6* mutations in disease progression, laboratories may wish to offer specific targeted analysis of *PHF6* for the monitoring of myeloid disease.

Finally, it is becoming clear that *PHF6* plays a role in lineage plasticity of hematopoietic malignancies, as *PHF6* mutations exist in T/Myeloid MPAL as well as MPAL, NOS (particularly B/T MPAL) and is frequently associated with early T-ALL which frequently shows myeloid marker expression. The underlying contextual factors, including cell of origin and cooperative gene mutations, remain to be elucidated to understand what drives a *PHF6*-associated malignancy to be T-lineage, myeloid lineage, or a combination of both.

## Author Contributions

OW conceived of the review subject matter and provided editorial review while JK primarily composed the manuscript. All authors contributed to the article and approved the submitted version.

## Conflict of Interest

The authors declare that the research was conducted in the absence of any commercial or financial relationships that could be construed as a potential conflict of interest.

## Publisher’s Note

All claims expressed in this article are solely those of the authors and do not necessarily represent those of their affiliated organizations, or those of the publisher, the editors and the reviewers. Any product that may be evaluated in this article, or claim that may be made by its manufacturer, is not guaranteed or endorsed by the publisher.
